# Evaluation of Dutch General Practitioners ultrasound referrals and opportunities for point-of-care ultrasound: A retrospective analysis

**DOI:** 10.1080/13814788.2025.2606572

**Published:** 2026-01-13

**Authors:** Jelien Geivers, Ralph T. H. Leijenaar, Lola Ramakers, Jochen W. L. Cals, Frank M. Zijta, J. Martijn Nobel, Ramon P. G. Ottenheijm

**Affiliations:** a Department of Family Medicine, Care and Public Health Research Institute, Maastricht University, Maastricht, The Netherlands; b Department of Radiology and Nuclear Medicine, Maastricht University Medical Center, Maastricht, The Netherlands; c Care and Public Health Research Institute (CAPHRI), Maastricht University Medical Centre, Maastricht, The Netherlands; d GROW-School for Oncology and Reproduction, Maastricht University Medical Centre, Maastricht, the Netherlands

**Keywords:** General practice, radiology, diagnostic imaging, abdominal ultrasound, point-of-care ultrasound, communication, referral

## Abstract

**Background:**

General practitioners (GPs) frequently refer patients for abdominal ultrasound. Depending on the clinical context, a ‘triage ultrasound’ can assess multiple potential causes of abdominal symptoms, while a ‘targeted ultrasound’ (point-of-care ultrasound, POCUS) focuses on specific indications (e.g. cholelithiasis).

**Objective:**

To assess whether medical questions posed by GPs in abdominal ultrasound referral letters are adequate for radiologists to perform their examination, and to identify indications for POCUS by GPs based on exclusion rates and alternative findings in radiological reports.

**Methods:**

Retrospective study analysing GP referral letters with corresponding radiology reports referred for abdominal ultrasound. Key variables: GP’s medical question, indication type and the radiologist’s final interpretation, following established diagnostic guidelines.

**Results:**

A total of 1,196 referral letters with corresponding reports were reviewed. Of these, 143 (12%) were excluded, primarily due to missing clinical information from the GP (102; 8.5%). The final sample comprised 1053 referral letters with reports (mean age 59.2 years; 60% female). Sixteen percent of referral letters lacked a medical question, and 33% included exclusively guideline-based indications. The most common guideline-based indications were urolithiasis (43%) and cholelithiasis (39%). For guideline-based requests, radiologists excluded the indicated condition in 75% of cases, and an alternative diagnosis was identified in fewer than 10%.

**Conclusion:**

GPs frequently provide insufficient clinically relevant information in abdominal ultrasound referral letters. Simple cases with well-defined clinical queries like cholelithiasis, urolithiasis, hydronephrosis and abdominal aortic aneurysm seem suitable for POCUS evaluation, as these are often excluded conditions for which the risk of overlooking serious diagnoses is low.

## Introduction

In the Dutch healthcare system, general practitioners (GPs) and radiologists work closely together to make informed health decisions for their patients. GPs serve as the first point of contact. When imaging is indicated, radiologists within radiology services primarily perform and interpret these examinations, documenting the findings in a structured radiology report upon the GP’s request. Abdominal ultrasound is a valuable diagnostic tool for assessing patients with abdominal issues. According to Vektis, an organisation compiling health insurance claims in the Netherlands, GPs request approximately 365,000 abdominal ultrasounds annually (data not publicly available), with diagnostic outcomes playing a crucial role in guiding patients’ healthcare journeys. In this collaboration between GP and radiologist, effective communication is key. On the one hand, the GP must provide the radiologist with comprehensive clinical information to achieve a high level of diagnostic accuracy [1]. On the other hand, uniform and structured radiological reporting featuring clear conclusions and recommendations enhances communication and interdisciplinary collaboration with the GP [2]. To enhance this interprofessional communication, the Dutch College of General Practitioners and the Dutch Society of Radiology have developed guidelines to optimise the use of abdominal ultrasounds referred to radiology services [3]. To our knowledge, no research has been conducted on the actual information provided by requesting GPs. Besides enhancing communication, these guidelines aim to reduce practice variation, streamline referrals and align diagnostic standards between primary and secondary care.

Ultrasound indications can be categorised as ‘triage examinations’ or ‘targeted examinations’. Triage ultrasounds are broad evaluations to assess a wide range of potential differential diagnoses, such as in patients with non-specific abdominal pain. In contrast, ‘targeted ultrasounds’, focus on simpler cases with specific, well-defined clinical queries, such as confirming a cholelithiasis [4]. In the Netherlands, radiologists typically perform triage ultrasounds for abdominal issues, regardless of the GP’s question. Dutch guideline-based indications, however, meet criteria for targeted ultrasound, requiring specific clinical queries, such as ‘Is there cholelithiasis?’ When performed by GPs, this is called point-of-care ultrasound (POCUS), offering real-time diagnostic support and guiding treatment decisions [5,6]. A Danish study showed POCUS improved diagnostic accuracy and changed treatment in 72% of cases [7]. Internationally, POCUS is gaining attention in general practice [7–11], but its use remains limited due to sparse evidence in guidelines, lack of standards and device costs. With growing evidence and affordable handheld devices, broader adoption is expected. Additionally, WONCA Europe recently recommended POCUS training for all GPs, adapted to local healthcare contexts [12]. Currently, about 5% of GPs in the Netherlands are registered as qualified abdominal ultrasonographers [13] and can perform these ultrasounds in their own practice, meaning the majority of GPs depend on radiology services.

In this evolving field, it is important to assess the efficiency, quality and clinical context of the current communication process between GPs and radiologists. Especially while exploring the potential benefits of shifting imaging responsibilities towards GPs. In a patient-centred healthcare system, optimising patient care at the most suitable location is essential, as efficient use of ultrasound diagnostics in general practice patients can enhance workflow efficiency, reduce unnecessary referrals and shorten the diagnostic process.

This study analysed the referral letter information and radiological reports of abdominal ultrasounds referred to a radiology department by the GP. The objectives were: (1) to assess whether the medical questions posed by GPs in abdominal ultrasound referral letters are adequate for radiologists to perform their examination, and (2) to better understand the potential role of POCUS in general practice, to identify ultrasound indications for POCUS by GPs based on exclusion rates and alternative findings in radiological reports.

## Methods

### Study design and population

With approval from the institutional review board, we conducted a retrospective analysis of GP-ordered abdominal ultrasound referrals and corresponding radiology reports at a large regional/academic hospital in the Netherlands, including cases from January 2023 to February 2024. Ultrasound referral letters and corresponding radiology reports were excluded if the patient was younger than 18 years or if the referral letter lacked both clinical information and a medical question from the referring GP.

#### Data extraction

We used a framework based on the guideline for abdominal ultrasound issued by the Dutch College of General Practitioners and the Dutch Society of Radiology to standardise data extraction from radiology reports [3]. According to this guideline, the GP referral letter should include at least a medical question, and the radiology report should consist of a descriptive part detailing the findings observed during the ultrasound, followed by a final interpretation or conclusion that considers the patient’s history, clinical condition and addresses the medical question provided by the GP. In line with this guideline, we included the following indications as guideline-based: urolithiasis, hydronephrosis, cholelithiasis, abdominal aortic aneurysm (AAA), hepatic steatosis and abdominal swelling, with the latter defined as urinary retention or inguinal hernia.

A single researcher (JG) extracted the type of medical question (absent, guideline-based, or non-guideline-based) and type of guideline-based indication from the ultrasound referral letters, and the radiologist’s final interpretation from the corresponding radiological reports. Further analysis focused on guideline-based indications in search for potential POCUS indications. Radiologists’ final interpretation was classified as: no abnormality, abnormality, unchanged compared to prior imaging, alternative diagnosis, or secondary findings.

An alternative diagnosis was defined as a finding unrelated to the GP’s medical question, yet potentially explaining the patient’s condition. Secondary findings were incidental discoveries unrelated to the primary reason for the ultrasound [3,14].

Consensus meetings were held to address ambiguities in the referral letter and report interpretation. To validate the data extraction, 100 ultrasound referral letters and corresponding radiology reports were independently reviewed by a second researcher (LR) and compared with the initial results.

#### Outcome measures

First, we analysed the frequency and types of medical questions posed by the GP in their referral letters, which addressed clinical indications. Subsequently, we focused on guideline-based indications, potentially suitable for POCUS, evaluating their frequency by type, the proportion ruled out or confirmed by radiologists in their reports, and the frequency of alternative diagnoses or secondary findings. Finally, in case of alternative diagnoses, their anatomical location was assessed.

#### Sample size and statistical analysis

During the study, 4,396 abdominal ultrasound referral letters and corresponding reports were available. A power analysis indicated that a sample of 859 ultrasound referral letters with corresponding reports was required to achieve a 95% confidence interval with a 3% margin of error, assuming a conservative 50% proportion. Anticipating a 20% exclusion rate, we aimed to analyse approximately 1,200 referral letters with corresponding reports [15].

Descriptive statistics for numerical variables involved mean and standard deviation (SD), while for categorical variables, frequency counts and percentages were used. To validate the data extraction process, Cohen’s kappa values plus 95% confidence interval (95%CI) were calculated to evaluate the inter-rater variability [16–19]. Data were analysed using SPSS (IBM Corp, Armonk, NY, USA, version 28.0.1.1)

## Results

**Figure 1.**
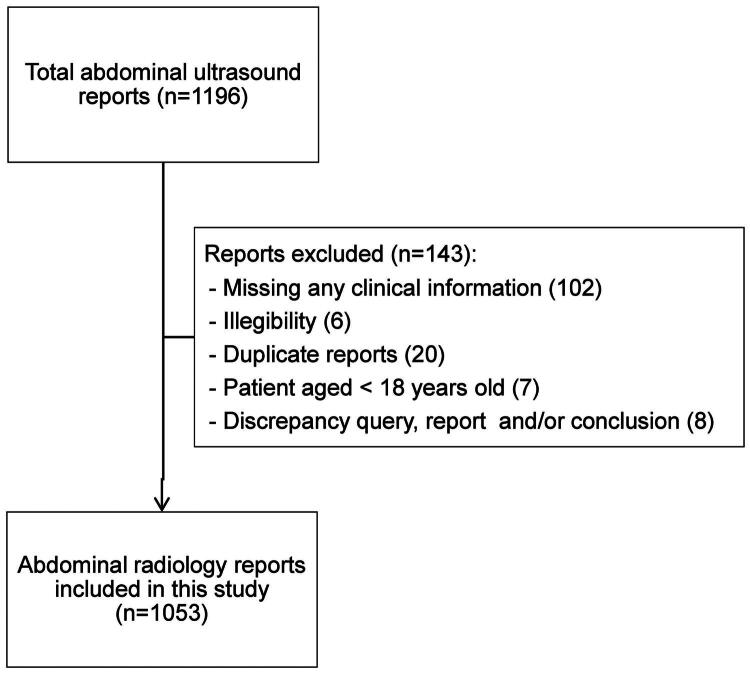
Flow chart of included referral letters and corresponding radiology reports.

A total of 1,196 ultrasound referral letters with corresponding reports were assessed for inclusion (Figure 1), of which 143 were excluded mostly due to missing information (*n* = 102), leaving 1053 referral letters with reports available for analysis. The mean age of patients was 59.2 years (SD 17.4), with 60% (*n* = 632) being female.

### Frequency and types of medical questions posed by the GP

**Figure 2.**
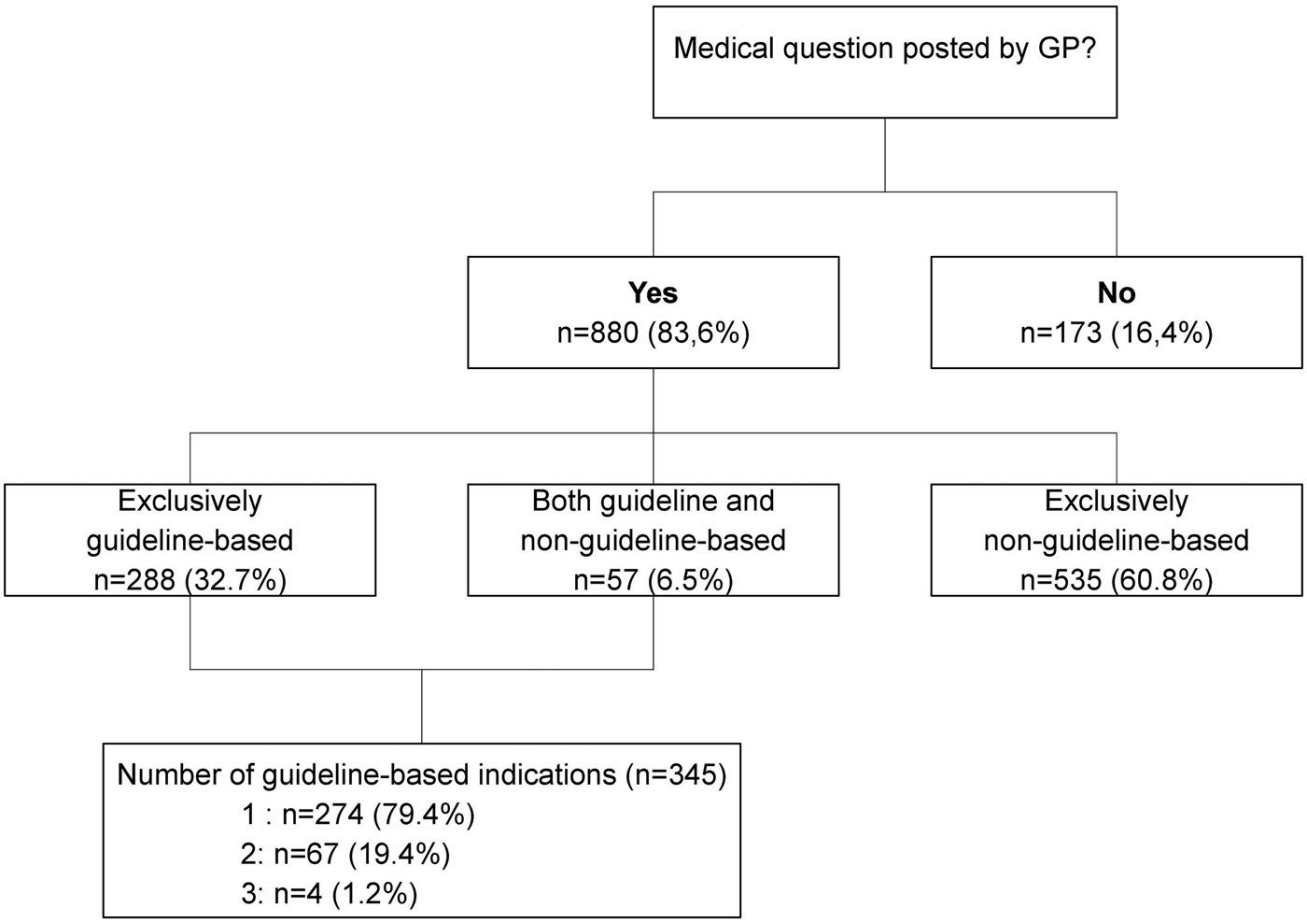
Frequency and types of medical question (indications) posed by GP in their referral letters (*n* = 1053).

Figure 2 shows the frequencies and types of medical questions provided by the GP. A total of 173 out of 1053 (16.4%) referral letters did not include a medical question. Of the 880 referral letters (83.6%) containing a medical question, 288 (32.7%) included exclusively guideline-based indications. Table 1 shows the frequency of each guideline-based indication. The most common guideline-based indications were urolithiasis (*n* = 148), cholelithiasis (*n* = 134), AAA (*n* = 56), and hydronephrosis (*n* = 44).

**Table 1.** Frequency of guideline-based indications for abdominal ultrasound within the group of guideline-based indications and the group of total referrals.

Indication	n*	% of reports with guideline-based medical questions (*n* = 345)	% of total radiology reports (*n* = 1053)
Urolithiasis	148	43.0	14.1
Cholelithiasis	134	38.8	12.7
AAA	56	16.2	5.3
Hydronephrosis	44	12.8	4.2
Hepatic steatosis	20	5.8	1.9
Urinary retention	12	3.5	1.1
Inguinal hernia	6	1.7	0.6

AAA, abdominal aortic aneurysm.

*Multiple indications per referral letter may be present, so summing up these indications exceeds the sample size (n) in the other columns.

### Radiologist’s interpretation in case of only guideline-based indications

**Figure 3.**
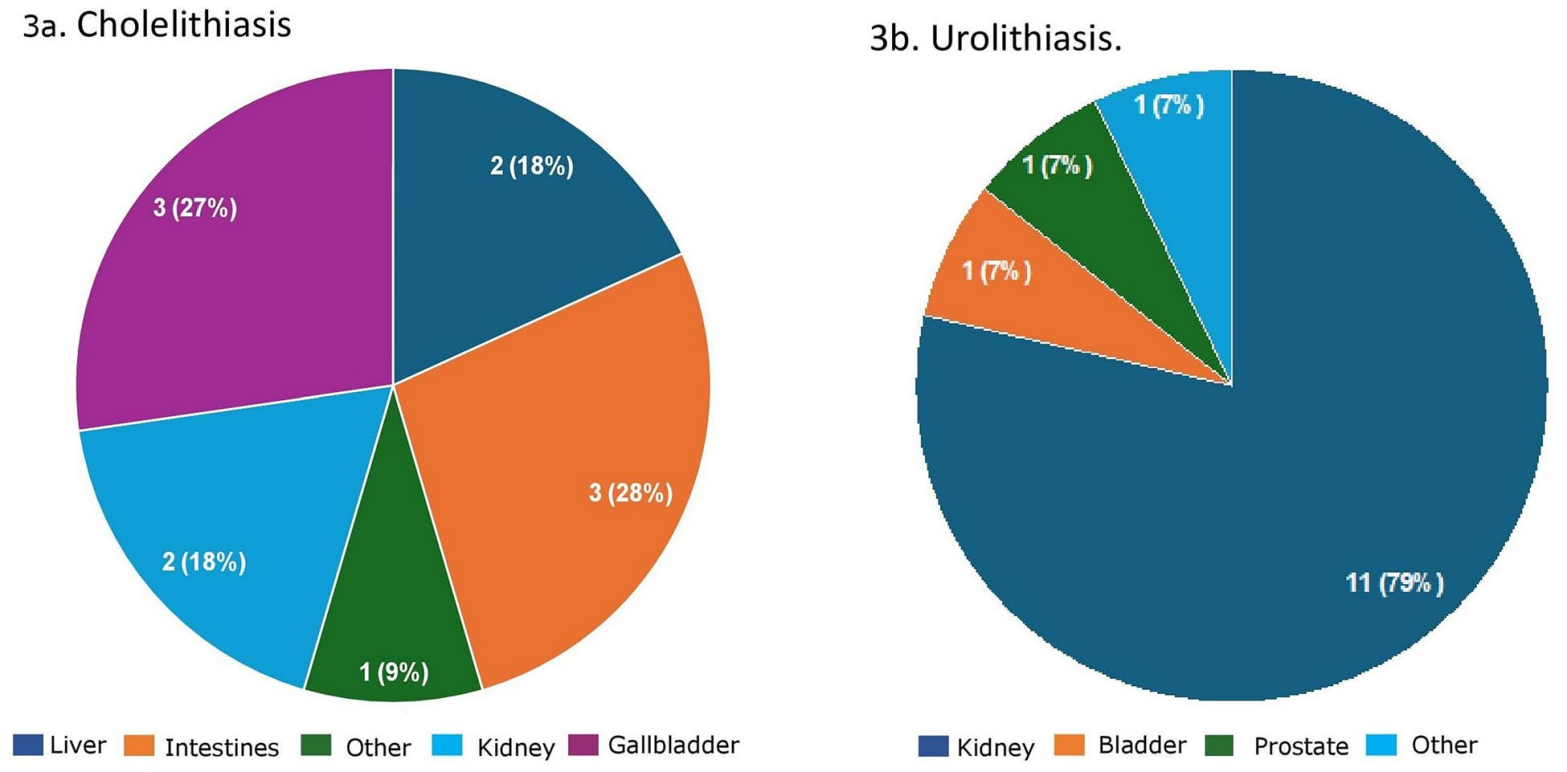
Organ or anatomical structure of the alternative diagnosis for (a) cholelithiasis (*n* = 11), and (b) for urolithiasis (*n* = 14) in reports with only guideline-based indications for abdominal ultrasound.

Overall, of all referral letters with only guideline-based indications (*n* = 288), those potentially suitable for POCUS, the indication was ruled out in 215 radiology reports (74.7%) and confirmed in 73 reports (25.3%). Additionally, an alternative diagnosis was present in 27 reports (9.4%), and secondary findings were present in 101 reports (35.1%). Table 2 provides an overview of individual indications. In 11 reports (3.8%) an alternative diagnosis was identified outside the gallbladder, kidney, or aorta, despite the GP’s medical question being specifically focused on these organs or structures. For the indication cholelithiasis, 55% of alternative diagnoses (6 out of 11) were located outside the gallbladder or kidney, while for urolithiasis, 21% of alternative diagnoses (3 out of 14) were outside the kidney (see Figure 3). For AAA, all alternative diagnoses (2 out of 2) were located outside the aorta. For hepatic steatosis, the alternative diagnosis was located within the liver. See the appendix for a complete overview, including descriptions and frequencies of alternative diagnoses (Table S1) and results for secondary findings (Table S2).

**Table 2.** Radiologist’s final interpretation per guideline-based indication for abdominal ultrasound with frequency of alternative diagnosis and secondary findings per indication in reports with only guideline-based indications (*n* = 288).

Indication	Total (*n*)*	Confirmed, *n* (%)	Ruled out, *n* (%)	Inconclusive, *n* (%)	Alternative diagnosis, *n* (%)	Secondary finding, *n* (%)
Urolithiasis	127	13 (10.2%)	107 (84.3%)	7 (5.5%)	14 (11%)	54 (42.5%)
Cholelithiasis	105	30 (28.6%)	74 (70.5%)	1 (1.0%)	11 (10.5%)	43 (41%)
AAA	52	8 (15.4%)	44 (84.6%)	0 (0%)	2 (4%)	11 (21.2%)
Hydronephrosis	34	9 (26.5%)	25 (73.5%)	0 (0%)	0 (0%)	12 (35.3%)
Hepatic steatosis	16	10 (62.5%)	6 (37.5%)	0 (0%)	1 (6.3%)	5 (31%)
Urinary retention	10	1 (10%)	8 (80%)	1 (10%)	0 (0%)	3 (30%)
Inguinal hernia	5	3 (60%)	2 (40%)	0 (0%)	0 (0%)	1 (20%)

AAA: abdominal aortic aneurysm.

*Multiple indications per referral letter may be present, so summing up these indications exceeds the number of referral letters (*n* = 288).

### Inter-observer variability

Agreement for categorising the clinical question posed by the GP was almost perfect (κ = 0.89; 95% CI 0.78–0.96). Similarly, almost perfect agreement was observed for the type of guideline-based clinical question (κ = 0.87; 95% CI 0.71–0.97) and non-guideline-based clinical question of the GP (κ = 0.88; 95% CI 0.78–0.96). For the final interpretation of the radiologist regarding guideline-based indications, agreement was substantial (κ = 0.74; 95% CI 0.64–0.82).

## Discussion

### Summary of main findings

Of all GP-requested abdominal ultrasounds, 16% contained no medical question in the referral letter. If referral letters lacking clinical information were included, this figure would rise to 24%, suggesting that in about a quarter of referral letters (essential) clinical information is missing. When the medical question was present, 33% of requests included only guideline-based indications, 61% featured only non-guideline-based indications, and 6% combined both. Urolithiasis, hydronephrosis, cholelithiasis and AAA were the most frequently requested guideline-based indications. When GPs limited their requests to guideline-based indications, radiologists could exclude them in approximately 75% of cases, identifying an alternative diagnosis in less than 10% of cases. Furthermore, in less than 4% of referral letters with guideline-based indications, potentially suitable for POCUS, the alternative diagnosis was unrelated to the examined organ or anatomical structure.

## Strengths and limitations

The main strength of this study lies in its retrospective design, providing insight into actual routine care by including a large number of GP-requested abdominal ultrasounds from a comprehensive database.

Approximately a quarter of the ultrasound referral letters lacked essential information, rendering them partly or fully unusable for analysis due to missing clinical information or containing only the clinical question posed by the GP. Nonetheless, this offers valuable insights into the quality of information GPs provide when requesting abdominal ultrasound imaging. Another possible limitation of this study is that data extraction was performed by a single researcher, which may have introduced interpretation bias and an increased risk of human error. However, we enhanced reliability and reduced potential bias through consensus-building and assessed the inter-observer variability with a second researcher, which demonstrated that there was substantial to perfect agreement.

## Comparison with existing literature

Ultrasound is a potentially valuable diagnostic tool for GPs, yet few studies examine GP-radiologist collaboration, focusing mostly on report content and structure [20]. Research on specific indications for POCUS in general practice is limited and varies across countries [7,8,11,21]. Some international consensus studies exist, but the evidence is sparse [10,21]. The guidelines for abdominal ultrasound issued by the College of General Practitioners and the Dutch Society of Radiology, used in our study as a framework, represent roughly half of all radiological diagnostic referrals from general practice, aligning with our findings [3]. Another Dutch study reported cholelithiasis as a requested indication in 25% of cases, higher than the 13% observed in our study, while urolithiasis was less common in their study at 6%, compared to 14% in ours [22]. Five previous studies on abdominal ultrasound requested by GPs showed abnormalities in 25–30% of the examinations, which is comparable with the 25% of abnormalities we observed in our study [22–26]. Compared to the other Dutch study, the diagnostic outcomes in both studies were similar, with cholelithiasis present in 10% versus 9% and kidney stones in 5% versus 2% [22]. Also, the frequency of an alternative diagnosis is comparable to another study (9 vs 8%) that included patients referred by a GP [25].

## Implications

The Dutch radiological guidelines aim to standardise referrals, improve communication and align diagnostic standards between primary and secondary care [3]. A Dutch-Belgian study highlighted consensus among radiologists and clinicians on providing adequate clinical information and clear questions in imaging referral letters [2]. Our findings reveal persistent challenges such as missing clinical details and medical questions, hindering radiologists’ ability to correlate ultrasound findings to patients’ clinical presentation. While patient interaction offers some guidance, radiologists often lack case-specific information and precise direction. Future studies and interventions aimed at optimising GP-ordered ultrasound examinations should address these issues. Notably, over half of GP requests cite indications not covered by the guidelines, reflecting their relevance despite exclusion from commonly listed indications. Clear definitions of POCUS indications are crucial if performed by GPs, as well as standards concerning skills and quality assurance. The increasing availability of handheld and affordable diagnostic ultrasound equipment makes ultrasound imaging accessible for non-radiologists, including the GP. Proper POCUS training of GPs is crucial to minimise diagnostic errors and ensure patient safety [12]. Our retrospective findings suggest that POCUS may be well-suited for evaluating cholelithiasis, urolithiasis, hydronephrosis and AAA, as these conditions are generally safe to assess with minimal risk of missing serious diagnoses. Experienced radiologists performed ultrasounds in this study. Conditions like hepatic steatosis and inguinal hernia remain challenging even for radiologists, and are excluded from emergency and internal medicine POCUS guidelines [27,28]. All these indications align with POCUS recommendations from the American Academy of Family Physicians and several European consensus studies [8,29,30]. In contrast, Dutch guidelines advise against using ultrasound for chronic abdominal pain or conditions like appendicitis and pancreatitis, citing diagnostic complexity and the need for specialist referral [3]. Future studies should prospectively assess the diagnostic and therapeutic yield of abdominal POCUS in this setting.

## Conclusion

GPs often provide insufficient clinically relevant information in their referral letter when requesting abdominal ultrasounds. Single and focused indications such as cholelithiasis, urolithiasis, hydronephrosis and AAA seem suitable for POCUS, as these are often excluded conditions for which the risk of overlooking serious diagnoses is low.

## Supplementary Material

Supplemental Material
