# Supplementary material for: Evaluation of Dutch General Practitioners ultrasound referrals and opportunities for point-of-care ultrasound: A retrospective analysis
Source: Eur J Gen Pract. 2026 Jan 13;32(1):2606572. doi: 10.1080/13814788.2025.2606572 (PMC12802515; doi:10.1080/13814788.2025.2606572)
Supplement: Supplemental Material [file IGEN_A_2606572_SM3883.docx]

**Appendix**

Table S1. The organ or structure location of **the alternative diagnosis** for the type of POCUS indication

| POCUS indication | Organ or structure | n | Alternative diagnosis |
| --- | --- | --- | --- |
| Gallstones |  | 11 |  |
|  | Liver | 2 (18%) | Hepatic steatosis (n=2) |
|  | Gallbladder/bile ducts | 3 (27%) | Sludge in gallbladder (n=2), and gallbladder polyps (n=1) |
|  | Kidneys | 2 (18%) | Hydronephrosis and nephrolithiasis (n=1), hydronephrosis (n=1) |
|  | Intestines | 3 (27%) | Diverticulitis (n=1), colitis colon ascendens (n=1), and severe constipation (n=1) |
|  | Other category | 1 (9%) | Induration of the muscles |
|  |  |  |  |
| Urolithiasis |  | 14 |  |
|  | Kidneys | 11 (79%) | Kidney tumor (n=1), hydronephrosis (n=8), lesion kidney (n=1), and kidney cyst with dilatation (n=1) |
|  | Bladder | 1 (7%) | Diverticula of the bladder |
|  | Prostate | 1 (7%) | Benign prostate hypertrophy |
|  | Other category | 1 (7%) | Tendinomyogenic |
|  |  |  |  |
| AAA |  | 2 |  |
|  | Lymph nodes abdominal/inguinal | 1 (50%) | Benign abdominal lymph node |
|  | Intestines | 1 (50%) | Severe constipation |
|  |  |  |  |
| Hepatic steatosis |  | 1 |  |
|  | Liver | 1 (100%) | Severe liver cyst |

AAA, abdominal aortic aneurysm; POCUS, point-of-care ultrasound.

Table S2. The organ or structure location of **the secondary finding** for the type of POCUS indication

| POCUS indication | Level structure/organ | n | Secondary finding |
| --- | --- | --- | --- |
| Inguinal hernia |  | 1 |  |
|  | Prostate | 1 (100%) | Benign prostate hypertrophy |
|  |  |  |  |
| Urinary retention |  | 3 |  |
|  | Kidneys | 1 (33%) | Nephrolithiasis and kidney cyst |
|  | Uterus | 1 (33%) | Uterus myoma |
|  | Liver | 1 (33%) | Hepatic steatosis |
|  |  |  |  |
| Hepatic steatosis |  | 5 |  |
|  | Pancreas | 2 (40%) | Lipomatosis pancreas |
|  | Liver | 1 (50%) | Hepatic steatosis |
|  | Gallbladder/bile ducts | 2 (40%) | Cholecystolithiasis (n=1) and gallbladder polyps (n=1) |
|  |  |  |  |
| Hydronephrosis |  | 12 |  |
|  | Liver | 2 (17%) | Simple liver cyst (n=1) and hepatic steatosis (n=1) |
|  | Kidneys | 8 (67%) | Simple kidney cyst(s) (n=6), angiomyolipoma (n=1), kidney atrophy and horseshoe kidney (n=1) |
|  | Prostate | 1 (8%) | Benign prostate hypertrophy with wall thickening of the bladder |
|  | Gallbladder/bile ducts | 1 (8%) | Cholecystolithiasis |
|  |  |  |  |
| AAA |  | 11 |  |
|  | Liver | 3 (37%) | Mild hepatic steatosis (n=2) and simple liver cyst (n=1) |
|  | Prostate | 1 (9%) | Benign prostate hypertrophy |
|  | Aorta abdominalis | 5 (45%) | Abdominal aortic atherosclerosis (n=4), atherosclerosis with stenosis of the iliac common artery (n=1) |
|  | Pancreas | 1 (9%) | Lipomatosis pancreas |
|  | Other category | 1 (9%) | Multiple cysts in small pelvic region |
|  |  |  |  |
| Cholelithiasis |  | 43 |  |
|  | Liver | 26 (60%) | Simple liver cysts (n=8), hepatic steatosis (n=17) and hemangioma (n=1) |
|  | Kidneys | 7 (16%) | Simple kidney cysts (n=6) and benign kidney tumor (n=1) |
|  | Intestines | 2 (5%) | Severe constipation (n=1) and signs of Crohn disease (n=1) |
|  | Pancreas | 3 (7%) | Lipomatosis pancreas |
|  | Abdominal aorta | 3 (7%) | Abdominal aorta atherosclerosis |
|  | Gallbladder/bile ducts | 1 (2%) | Gallbladder polyps |
|  | Spleen | 1 (2%) | Calcifications (sign of tuberculosis) |
|  |  |  |  |
| Urolithiasis |  | 54 |  |
|  | Kidneys | 26 (48%) | Simple kidney cysts (n=23), benign kidney tumor (n=1), angiomyolipo-  ma (n=1) and horseshoe kidney (n=1) |
|  | Liver | 11 (20%) | Hepatic steatosis (n=8) and simple liver cysts (n=3) |
|  | Prostate | 2 (4%) | Benign prostate hypertrophy |
|  | Bladder | 1 (2%) | Bladder lesion |
|  | Pancreas | 3 (6%) | Pancreatic lipomatosis (n=3) |
|  | Adnexal | 3 (6%) | Adnexal cysts (n=3) |
|  | Intestines | 4 (7%) | Diverticulosis/diverticulitis (n=2), constipation (n=1) and signs of Crohn disease (n=1) |
|  | Aorta abdominalis | 2 (4%) | Abdominal aorta atherosclerosis |
|  | Spleen | 1 (2%) | Calcifications (sign of tuberculosis) |
|  | Other category | 1 (2%) | Mesenterial cyst |

AAA, abdominal aortic aneurysm; POCUS, point-of-care ultrasound.
